# Can hospital promotional activities be more ethical?

**DOI:** 10.12669/pjms.303.5111

**Published:** 2014

**Authors:** Yiyi Chen, Zhou Yin, Qiong Xie, Zhexin Shao

**Affiliations:** 1 Yiyi Chen, Hospital Office, The First Affiliated Hospital, College of Medicine, Zhejiang University, 79 Qingchun Road, Hangzhou, 310003, Zhejiang, China.; 2 Zhou Yin, Clinical Laboratory, The second Affiliated Hospital of Zhejiang Chinese Medical University, 316 Chaowang Road, Hangzhou, 310014, Zhejiang, China.; 3 Qiong Xie, Information Centre, The First Affiliated Hospital, College of Medicine, Zhejiang University, 79 Qingchun Road, Hangzhou, 310003, Zhejiang, China.; 4 Zhexin Shao, Hospital Office, The First Affiliated Hospital, College of Medicine, Zhejiang University, 79 Qingchun Road, Hangzhou, 310003, Zhejiang, China.

**Keywords:** Professional Ethics, Publicity, Hospital

## Abstract

At present, there exist a lot of violations of medical ethics in advertising and promotional activities, which have been infringing the rights of patients. Therefore, the ethical criteria should be established as soon as possible to regulate the hospital promotional activities, to regain the trust of people.

## INTRODUCTION

From the marketing perspective, hospitals are typical service providers.^1^ In some countries, with the development of market reform, the “do not advertise principle” has been abolished, and marketing activities seem to be widespread in hospital surroundings and some marketing also goes on in GP practices.^2^ Many hospitals have fallen back on publicity stunts, through television, radio, newspapers, magazines and other promotional media to increase market competitiveness. In order to maximize the advertising value, many hospitals are showing different degree of moral failings and violation of ethical standards in promotional activities, all of which have severely damaged the image of hospitals.

**LACK OF ETHICS IN PUBLICITY**

Any promotional activity should be based on the truth, but in fact, many hospitals have ignored this and deviated from the direction of the nature of publicity, paying more attention to the marketing strategy. Some promotional materials were too exaggerated in literary expression, and there existed too many words of fulsome praises, even false or misleading claims ignoring the principle of maintaining patients record confidential. On the other hand, some hospitals illegally revealed patient's personal information such as identity, therapeutic schedule, disease outcome and so on, just to prove the authenticity of material. All this has caused great harm to patient’s spirit, and brought great inconvenience in their daily life.

In order to capture the new medical market, some hospitals hurriedly declared grasping the latest technology that had not yet accumulated successful experience, and marketed their services that had not been fully validated in clinical trials. Due to the lack of medical knowledge, patients had no ability to distinguish the true from the false, and would be easily misled and suffered economic and spiritual loss.

Some hospitals frequently use television or internet to broadcast surgical operation, and the live broadcasting had gained worldwide popularity.^3^ But these activities associated with filming would disrupt the surgeon's attention and increase the rate of surgical errors or the risk of peri-operative infection associated with increasing numbers of people in an operating room, particularly if they were unfamiliar with aseptic technique.

The image of hospitals in recent years has presented basically in a positive way in mass media, but the negative reports about hospitals keep increasing. Due to fears of negative media coverage about medical dispute or medical negligence, hospitals had an instinctive psychological rejection for a variety of media interviews, therefore tended to use the diplomatic language like "no comment". The public felt their right to know was not respected. Since hospital refused to provide relevant information, they had to turn to other channels (more to the patient's side). The hospital's escape behaviors would easily lead to dissatisfaction and criticism of society.

**ETHICAL MEASURES**

All those above mentioned actions had been undermining trust in hospitals. It's essential for hospitals to know how to improve the service quality and uphold professional ethics in publicity while dealing with the crisis of confidence. Hospital should pay more attention to the ethical issues, such as methods, orientation, basic rules and purposes of publicity, in addition to improve the quality of medical treatment or medical service ability. As one of the necessary means to regain the trust of the public and expand the hospital reputation, publicity must ensure to use "truth" as base line of moral, "objective" as value pursuit and “accuracy” as standard of publicity. As a moral principle, publicity ethics must be followed in all hospital's promotional activities.

Mass media has important influence on the public understanding of science, medical practice and ethical controversies in health care.^4^ It also becomes evident that the success of hospital publicity should be based on adapting to patients' needs, minimizing the uncertainties and risks experienced by the patients and maximizing the perceived service quality.^1^ Therefore, hospital should pay particular attention to the means of publicity and the universality of the promotional content appearing in the mass media, make an honest description of key characteristic faculties, new techniques and service projects, and explain to the public in simple easy to understand language or case which is most close to public life. Publicity materials related to patients' right of health and privacy must receive the review of ethics committee. Particularly, live broadcast of operation in the modern media must accept professional evaluation about potential impact on patients. Surgeons should pay special attention to the needs and rights of the potential patient-subject before recording or broadcasting an operation.

Hospitals also play a very important role in public health emergency surveillance and early warning. Public health emergencies endangering public life and health always started imperceptibly. Such incidents closely relating with most people's health usually come into people's vision in a sudden way, often cause people worry and even social panic. As the main force in early warning and alleviating social panic, hospital should establish a spokesperson system to deal with the growing challenges in communicating with the public.

Citizens have the right to know and to monitor the hospital activities. Especially, patients have the right to obtain information related to their own vital interests, so hospitals must satisfy the public demands in this area, and abandon diplomatic language such as "no comment", to adopting positive attitude of not concealing, no shirking and no quibbling toward problems and hot spot topics, and actively take face-to-face contact with the media to tell the truth. Improving the doctor-patient communication to create a harmonious relationship is the characteristic of health care industry. These will help create more open and considerate social atmosphere for all people ensuring a bright and reliable medical prospects.

## CONCLUSION

The rise of new propaganda technologies and media is a new chance and a new challenge, but the ethical issues remain the same.^5^ The hospital should focus on patients' interests to enhance the management of publicity, & build a harmonious medical environment, to restore the trust of patients to hospital. Especially, in today's China, major medical institutions are state-owned public hospitals, which are different from commercial organizations, so, more attentions should be paid to social benefits. Establishing more appropriate appraisal procedures and restriction mechanisms for publicity activities (especially advertising) meets the ethical requirements.
